# Diagnosing Spinal Gout: A Rare Case of Back Pain and Fever

**DOI:** 10.1155/2021/7976420

**Published:** 2021-09-29

**Authors:** Andres Cordova Sanchez, Maneesh Bisen, Farzam Khokhar, Adriana May, Jihad Ben Gabr

**Affiliations:** ^1^Department of Medicine, SUNY Upstate Medical University, Syracuse, NY 13210, USA; ^2^Department of Pathology, SUNY Upstate Medical University, Syracuse, NY 13210, USA; ^3^Division of Rheumatology, SUNY Upstate Medical University, Syracuse, NY 13210, USA

## Abstract

Gout is a common inflammatory arthritis that has a high prevalence worldwide. It is characterized by monosodium urate deposition, usually affecting the joints and soft tissue of the lower extremities. Urate deposition in the axial skeleton resulting in spinal gout is rare. However, it seems to be more prevalent than usually thought, largely because it is underdiagnosed. Imaging findings are, for the most part, nonspecific and often mimic infectious etiologies. Definitive diagnosis requires pathological examination. Thus, it can be easily missed. We present a 41-year-old male with a seven-year history of untreated gout who came in with severe back pain, fevers, and radiculopathy. He was initially diagnosed with vertebral osteomyelitis. However, after a biopsy, spinal gout was confirmed. Spinal gout can be misdiagnosed as vertebral osteomyelitis given the similarities in presentation and imaging findings. This case report highlights the importance of keeping spinal gout as a differential of vertebral osteomyelitis, especially in patients with long-standing or uncontrolled gout with tophi.

## 1. Introduction

Gout is the result of an inflammatory response to monosodium urate crystal deposition in the body. It has a prevalence of approximately 4% in the US, significantly higher in men than in women 5.9% vs 2% [1]. Alteration of serum urate is primarily related to an imbalance in its production and excretion. Several factors have been associated with increased serum urate levels: poor dietary patterns, obesity, hypertension, metabolic syndrome, type 2 diabetes mellitus, chronic kidney disease, and medications are the most commonly cited [2].

Gout typically presents as acute mono, oligo, or polyarticular arthritis, most commonly affecting sites of the midfoot, ankle, and knee joints. Typically, flares have a rapid onset and can present with fevers, joint swelling, and restricted movement of the joint. Laboratory testing can show nonspecific signs of inflammation such as elevated C-reactive protein, platelet, and neutrophil counts. If untreated, chronic inflammation may result in a granulomatous inflammatory response with tophi formation. Due to their similarity, septic arthritis is an important differential diagnosis of an acute gout attack and should always be ruled out. Therefore, synovial fluid analysis is the gold standard for diagnosis [2].

Spinal gout is considered a rare condition. However, some studies have reported an estimated prevalence of 22% to 35% [3, 4], but given the wide range of symptoms and lack of awareness, its diagnosis can be challenging. When present, the lumbar spine is the most commonly affected [3, 5].

We present a case of spinal gout that, due to its initial clinical, laboratory, and imaging evaluation, was thought to be vertebral osteomyelitis and subsequent tissue biopsy led to a definitive diagnosis.

## 2. Case

A 41-year-old male with a past medical history of gout (diagnosed seven years ago, not on urate lowering therapy with a total of nine flare episodes since diagnosis), uric acid nephrolithiasis, and hypertension on lisinopril, who was transferred from an outside facility with a diagnosis of septic arthritis secondary to vertebral osteomyelitis.

**Figure 1:**
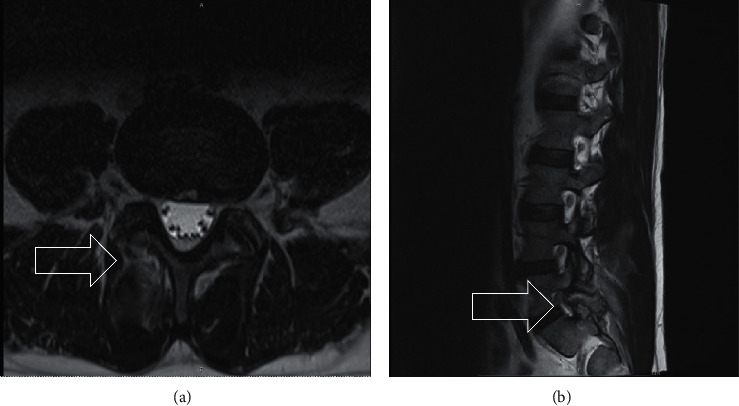
(a) T2 weighted axial image at L5-S1 level; (b) T2 weighted sagittal image. There is moderate diffuse circumferential disc bulging, severe right facet arthropathy, marked inflammatory changes involving the right facet joint at L5-S1, and significant edema in the posterior right paraspinous musculature near the lumbosacral junction.

The patient presented with lower back pain with right-sided radiculopathy of one-month duration. Two weeks before admission, he developed pain and swelling of both elbows and knees. His primary care physician prescribed a prednisone taper regimen which he completed one week before admission. The pain initially improved with prednisone. However, once the prescription had finished, he developed excruciating back pain with fevers, prompting him to go to an outside hospital, where the diagnosis of vertebral osteomyelitis was made based on an MRI of the spine showing right facet arthropathy and marked inflammatory changes at L5-S1 (Figures 1(a) and 1(b)). He was placed on broad spectrum antibiotics and transferred to our institution.

**Figure 2:**
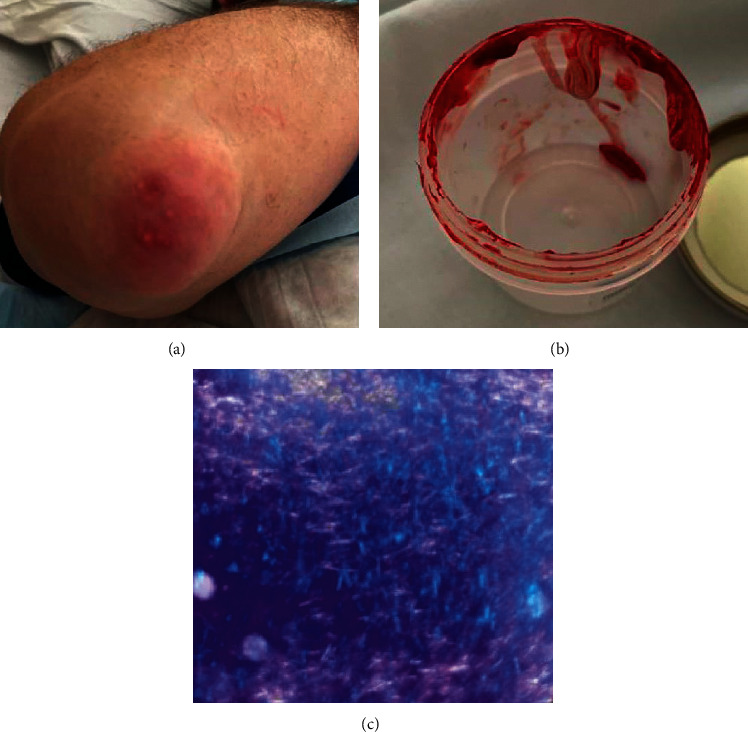
(a) Tophi involving the right elbow; (b) white, chalky deposit from aspirate; (c) monosodium urate monohydrate (MSU) crystals from a gouty tophus viewed under polarized light. Strong birefringence was noted along with the needle-shaped morphology classic for MSU.

On physical exam, he was overweight with a body weight max index of 28.5 kg/m^2^, tachycardic at 104 beats per minute, hypertensive 153/102 mmHg, and tachypneic 24 respirations per minute. Bilaterally, his knees, elbows, and right third proximal interphalangeal and metacarpophalangeal joints were warm, tender, and swollen. Bilateral elbows showed the presence of swollen, bulbous white growths under the skin, likely representing tophi in the setting of uncontrolled gout (Figure 2(a)). The patient underwent bedside aspiration of the right elbow which revealed a white, chalky deposit (Figure 2(b)). The aspirate was visualized under light microscopy, confirming the presence of monosodium urate crystals (Figure 2(c)).

Laboratory results were significant for leukocytosis WBC 12 10 *∗* 3/uL, ESR 85 mm/hr, CRP 154 mg/L, and elevated uric acid level at 8.7 mg/dl. Blood, urine, and joint cultures were negative.

**Figure 3:**
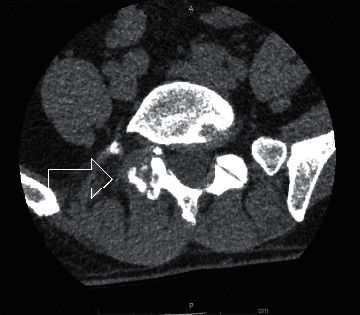
CT of the lumbar spine showing erosive degenerative changes of the right L5-S1 facet joint (arrow), with mild soft tissue prominence surrounding this region. Narrowing of the right L5-S1 neural foramina is also appreciated.

**Figure 4:**
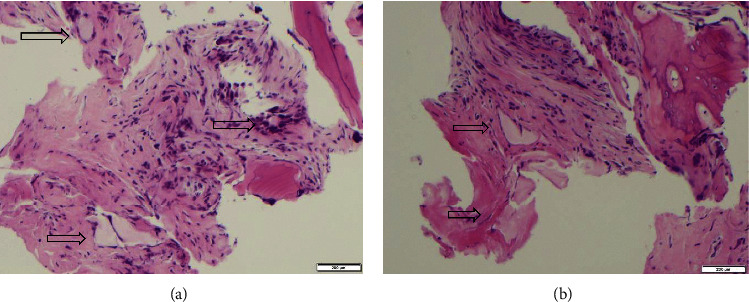
(a, b) Biopsy from right L5-S1 facet joint demonstrating large amorphous deposits (arrows) surrounded by granulomatous inflammation and foreign body giant cells, consistent with spinal gout.

CT spine showed erosive degenerative changes at L5-S1 facet joint with hypertrophy (Figure 3). Despite antibiotic therapy, he continued to have significant pain, swelling, and fevers and subsequently underwent right L5‐S1 facet joint biopsy (Figures 4(a) and 4(b)), confirming the diagnosis of spinal gout. Colchicine and allopurinol were started with significant symptom improvement. He was discharged with plans to modify his allopurinol dose as an outpatient to achieve a goal of uric acid <5 mg/dL.

## 3. Discussion

Spinal gout might be more common than thought [3, 4], but due to lack of awareness, it can easily be misdiagnosed. It is unclear what percentage of patients are asymptomatic [5]. There are few studies addressing its prevalence, where it has been reported in up to one-third of patients with gout [3]. Back pain is the most common presenting symptom, followed by neurologic symptoms due to spinal or nerve root compressions [3–5].

Imaging is, for the most part, nonspecific in the diagnosis of spinal gout. When present, X-ray findings appear to correlate with extensive lesions [5]. A review of the literature found that MRI with contrast was able to identify correctly a tophaceous lesion only 21% of the time [5]. MRI findings can mimic those found in osteomyelitis.

In the 2015 gout classification criteria, the evidence of urate deposition in ultrasound or dual energy CT (DECT) of a joint that is or has been symptomatic is considered one of the criteria [6]. The ultrasound finding (double contour sign) is unlikely to be useful in the spine [7]. DECT is able to identify materials based on how they absorb two different photon energy levels [8]. Its sensitivity and specificity are 0.79–0.93 and 0.75–0.9 with 95% confidence interval, respectively [9]. DECT is a relatively new diagnostic tool that could potentially aid in the diagnosis and eliminate the need for tissue diagnosis. However, this technology is not yet widely available and has not been validated to be used in the spine as a substitute for biopsy confirmation.

CT is better than MRI for the detection of spinal gout. The characteristic findings are articular erosions and sclerotic margins with increased surrounding density [7]. Lumezanu et al. have proposed that in the presence of characteristic CT findings with proven gout, biopsy is not needed for the diagnosis [7].

Similar to an acute gouty attack of any other joint, spinal gout can be misdiagnosed with vertebral osteomyelitis. Both can present with severe back pain, fever, and elevated acute phase reactants and can have undistinguishable imaging findings. Acute gouty arthritis can present with systemic inflammatory response syndrome (SIRS) and can be misdiagnosed as sepsis [10]; this finding has also been described in spinal gout [11]. Janssens et al. created a diagnostic tool to differentiate severe gouty arthritis from sepsis in the primary care setting [12]. This tool could be useful in hospitalized patients too [13]. Although MRI is the modality of choice for vertebral osteomyelitis. Special consideration should be given to patients with gout, particularly with long-standing, poorly controlled disease with hyperuricemia or the presence of tophi. In these cases, CT and tissue biopsy can be used to aid in the diagnosis.

Our patient came in with initial suspicion of sepsis secondary to vertebral osteomyelitis. This diagnosis was made given suggestive MRI findings, elevated CRP and ESR, and clinical presentation of severe back pain with fever, tachycardia, tachypnea, and leukocytosis. Given the negative infectious workup and lack of response to antibiotics, spinal gout became high on the differential. The patient had acute polyarticular arthritis in other joints, visible tophi on both elbows with MSU crystals seen under the microscope. Subsequent spinal biopsy further confirmed the diagnosis.

When performing tissue diagnosis in suspected spinal gout, it is important to remember that uric acid crystals are water soluble and are destroyed by formalin fixation. In formalin fixed tissues, gouty tophi appear as large amorphous deposits in the dermis and subcutaneous tissues surrounded by granulomatous inflammation and foreign body giant cells (Figures 4(a) and 4(b)). In our case, we could not demonstrate monosodium urate crystals due to the tissue samples being placed in formalin.

The mainstay therapy in acute gouty attacks is pain relief with nonsteroidal anti-inflammatories, colchicine, and corticosteroids. For patients presenting with symptomatic spinal gout and no focal neurological deficit, it appears that medical therapy alone is successful in managing symptoms [5]. Our patient had resolution of joint and back pain days after colchicine was prescribed. We suspect that the steroids prescribed previous to admission decreased inflammation, explaining the subacute onset of symptoms.

## 4. Conclusion

Spinal gout is encountered more often than usually perceived. It can present with an acute gouty attack and can be confounded as septic osteomyelitis. It is crucial to think of it as a differential diagnosis of vertebral osteomyelitis in patients with a history of gout, especially if they have advanced disease. Tissue diagnosis is of vital importance to make an accurate diagnosis. Initial treatment involves symptom management with NSAIDs, colchicine, steroids, and urate lowering therapy.

## Data Availability

No data were used to support this study.
